# Icing or cake? Grant competitions as a model for funding chronic disease prevention in Tasmania, Australia

**DOI:** 10.1093/heapro/daac115

**Published:** 2022-09-27

**Authors:** Victoria Loblay, Kate Garvey, Alan Shiell, Shane Kavanagh, Penelope Hawe

**Affiliations:** The Australian Prevention Partnership Centre, based at Menzies Centre for Health Policy and Economics, Sydney School of Public Health, Faculty of Medicine and Health, The University of Sydney, New South Wales, Australia; Youth Mental Health and Technology Team, Brain and Mind Centre, The University of Sydney, Sydney, New South Wales, Australia; Department of Health, Tasmanian Government, Hobart, Tasmania, Australia; School of Psychology and Public Health, La Trobe University, Melbourne, Victoria, Australia; School of Psychology and Public Health, La Trobe University, Melbourne, Victoria, Australia; School of Health & Social Development, Faculty of Health, Deakin University, Geelong, Victoria, Australia; The Australian Prevention Partnership Centre, based at Menzies Centre for Health Policy and Economics, Sydney School of Public Health, Faculty of Medicine and Health, The University of Sydney, New South Wales, Australia

**Keywords:** funding mechanisms, resources, community-centred grant making, sustainability, community capacity building

## Abstract

Competitive grant funding is a well-established mechanism for generating activity and interventions in the field of chronic disease prevention. Yet grant competitions may be burdensome for organizations, and money may not be enough to bring about lasting change in communities. In this study, we explore the dynamics of awarding and receiving money in the context of a state-level government grant competition to support community organizations and promote community-driven action for health and well-being in Tasmania, Australia. Drawing on reflections of successful grant recipients and real-time observation of grant decision-making, we consider the role and value of grant competitions both for individual organizations and for generating broader change processes. We found that grant competitions operated according to an ‘icing-on-the-cake’ approach to funding, whereby money was provided for extra activities and new initiatives. In this way, the grant competition was valuable not only for stimulating new programme activities but also to effect broader organizational change, such as developing planning capacity, igniting new directions and pushing organizations towards ‘health’-focused activities. But for smaller organizations, grant funding was often stretched to support core work (i.e. cake rather than icing). Grants targeting specific focus areas could be a drain on resources if they diverted staff time away from core activities. We suggest an alternative approach to funding in which grants are able to be more responsive to the needs of community organizations and the support they require, as well as to desired outcomes. We describe the policy response to the results to date.

## INTRODUCTION

The field of health promotion has a long history of using competitive grant funding to foster community-based health promotion programme delivery. Different types of organizations may award grant funding including government, philanthropic foundations and industry. Government funds are typically targeted to generate activity around one or more national or state policy priority areas (e.g. HIV prevention or physical activity) as an attempt to increase the population-level ‘dose’ of prevention (Caperchione *et al*., 2010; Tamminen *et al*., 2014). Hence, such grants are also referred to as categorical or specific-purpose grants, and can be distinguished from block grants, which are typically population based and allocated to more broadly defined activities such as community development or health promotion.

But competitive grant processes are not simply intended for the delivery of programmes to communities. They build capacity. Grant competitions can be re-purposed to provide financial and technical support to enable community-based coalitions to develop new programmes targeting specific groups and specific health problems (Wagner *et al*., 2000). In this way, competitive grant making can be thought of as a way to ‘amplify’ resources by giving a ‘jump-start’ to organizations (Orfield *et al*., 2015, p. 5). Winning competitive grants also carries symbolic value, signalling that organizations are worth investing in (Faulk, 2011), and grant competitions can trigger new funding patterns around a particular policy issue, helping it gain leverage (Breihan, 2009).

Grant processes have been studied in a variety of ways. Evaluations of grant processes are often conducted through interviews with grantees (Cass *et al*., 2004). Insights gained can be useful for local improvement (Hartwig *et al*., 2006). However, the benefit of external funding at the local level may vary according to what resources are already in place within a community (Kavanagh *et al*., 2022). Another factor influencing the impact of local grant processes may be the historical changes in the mix of funding mechanisms for the wider population and, in particular, the ratio of block grants to categorical grants (Institute of Medicine, 2012). This larger context may affect the degree to which local grant-funded health promotion activity might be expected to compensate for other mechanisms of funding being absent. This invites a more fine-grained understanding of the role and value of a grant in its context, and a better appreciation of where grant processes fit alongside other resources to support prevention and build prevention capacity. We set out to explore how grant competitions mobilize resources in communities. In particular, we wanted to understand this from both the ‘giver’ and ‘receiver’ sides. That is, we sought to understand how those involved in the granting process conceived of the grants in relation to the existing resources within organizations and/or communities, and how the grant funding was understood to ‘add’ to or catalyse these resources.

### Context

In 2019, the Tasmanian government launched a new fund to promote community-driven action for health and well-being. The fund has provided select community organizations with large and small grants of up to $200 000 over 2 years for projects addressing targeted health and well-being priority areas (e.g. healthy eating, mental health) with a focus on building community connections. The fund provided roughly AU$2 million in both 2019 and 2020.

Organizations had to apply for funding through single funding round each year, advertised 6 weeks ahead of the application deadline. Grant recipients were selected from those that applied on the basis of recommendations by a Review Panel made up of representatives from the Department of Health, Department of Education, Department of Communities Tasmania and Primary Health Tasmania. Meeting over 2 full days, the Review Panel assessed more than 100 applicants each funding round. Proposed projects were weighed against one another based on their individual merit, as well as considerations of the ‘mix’ of grants, that is, how funded projects complement one another and reach across different regions of the state. Applications were appraised against criteria including evidence base, the diversity or number of participants, access for priority communities, collaboration with other organizations and community partners, evaluation plans, and value for money. Projects were also required to demonstrate sustainability and had to describe how they would continue to provide benefits to communities beyond the lifetime of the grant.

## METHODS

This research is part of a broader study of funding processes in chronic disease prevention in Tasmania, conducted as part of a collaborative chronic disease prevention research partnership involving university-based academics and policy-makers (Wutzke *et al*., 2017). The findings reported here are based on a case-study approach using a range of data collection strategies to build a nuanced picture of the grants programme (Côté-Boileau *et al*., 2020). We drew on interviews with grant recipients, audio-recordings from Review Panel meetings and ongoing collaborative engagements with key policy-makers working with the funding body.

Successful grant recipients from the 2019 funding round were identified through funding body records and approached by us to participate. Of 21 successful grant recipients, 16 agreed to participate, 4 did not respond and 1 chose not to partake in the research. Participants were based in not-for-profit community organizations (*n* = 11) and local government organizations (*n* = 5). Half of the participants had received small grants up to $50 000 and half had received large grants up to $200 000. From May to August 2020, we conducted semi-structured interviews with participants for 60–90 minutes. Plans for face-to-face interviews were suspended due to nationwide COVID-19 lockdowns and interstate border closures, and all interviews were recorded over Zoom and transcribed verbatim. Interviews began with open-ended discussions about how grant programmes had changed during lockdown, which we report elsewhere in detail (Loblay *et al*., 2022). Participants were encouraged to tell their grant ‘story’, including details about the idea for the grant application, the process and time involved in putting together the application, and how the grant affected the existing activities undertaken within their organization and the work they wanted to do (see Appendix 1 on the Supplementary Materials.). They were also asked to reflect on the current funding structures supporting their organizations and the role of grants in leveraging resources and organizational capacity. Written and verbal consent was obtained for each interview.

Data from over 18 hours of Review Panel meetings were audio-recorded and transcribed verbatim. In the 2019 meetings, there were nine people present (seven decision-makers, two note-takers). There were nine people in the 2020 meetings (six decision-makers, three note-takers). All members of the review panels provided written consent. One of the authors (KG) adopted a role consistent with that of a ‘para-ethnographer’ (Vangkilde and Rod, 2020) providing the research team with reflexive observations about funding processes based on her experience as a policy-maker embedded within the funding body and as a member of the Review Panel. KG’s dual role also allowed for key analytical insights from the research to be directly relayed to the funding body for quality improvement of the grants processes, enabling better use of government funds.

All transcripts were anonymized and coded in NVivo 12 (2021) by one of the authors (VL). Initial thematic analysis (Braun and Clarke, 2006) was shared with the research team in monthly meetings where we engaged in collective analysis and interpretation of major themes, using select quotes and vignettes from the data set. Analysis from the 2019 review panel was synthesized, reported and discussed by panel members as part of the 2020 review panel meetings. This process of interpretive analysis was enhanced by contextual knowledge of research team members gained through additional interviews and fieldwork in the broader study, as well as the embedded experiential knowledge of the policy-maker.

## RESULTS

Successful grant recipients (GRs) spoke specifically about their recent experience and reflected generally about grant competitions as funding models. Many had long experience with grant competitions, observing that funding had become more competitive. Although the term ‘grants’ was sometimes used to refer to longer term recurrent funding arrangements (3 or more years), in the examples below, the term is used only in reference to short-term funding (2 years or less). We present the results first using the reflections of the GRs. We then introduce insights from the panel discussion, where we witnessed to-and-fro and real-time ‘agonizing’ on some of the same issues as the GRs.

### Reflections from grant recipient interviews

#### Grants assist organizational development

GRs described multiple ways grant competitions were of value for their organizations. For organizations that had secured recurrent funding from other government funds, this grants programme was a way to experiment with new initiatives that took them beyond their usual activities:

Our one-off projects, I always put them to the side. They’re the extra. They’re the icing on the cake… Our [grant from funding body], that’s 15,000. Again, that’s icing on the cake. It’s for extra.

One GR described her organization as ‘at that awkward adolescent phase of our grant management’ and thought they should apply for more grants because of the boost it would provide to the organization’s strategic development:

The more we apply for grants, the better the momentum builds, and we’re just kind of at the cusp of that right now… it would then be easier to do more.

In this way, the value of grants appeared to be contingent on the stage of an organization’s maturation. Another GR described how relationships built up over many years gave them an edge in grant competitions. People knew the GR could successfully run programmes and partnerships were crucial for future grant success.

For a GR in a local government organization, grants could push the organization beyond their traditional focus towards a ‘health’ approach:

The money is linked to that but it’s not all about the money… It’s much more about that sense of feeling enabled, encouraged, pushed to particularly follow a focus.

Some GRs described how grants could build capacity for programme planning. One GR recalled a previous experience with a different grant competition, where they were funded to do ‘preapplication work’ such as developing the programme idea and partnerships:

That [funding] was valuable because it enabled us to find really strong partners, and to really develop the plan properly, rather than just going with okay, here’s a million dollars available on grants, let’s just come up with something quickly and put it in.

Another organization, a well-established leader in their field, no longer needed grants to deliver their services. They planned to use their grant to build the case for expanded core funding:

We did the pilot, we built some evidence around the effectiveness of the pilot. Now, we’re able to do a larger scale piece of work. We’re hoping that, again, this continues to build the foundation of why this is so important, in the hope that we can make a case that this could get ongoing funding from government.

This illustrates the importance of thinking through how grant money is distributed in relation to what is already happening within an organization as well as the resources that are necessary to plan for a new programme of work with a community.

#### Grants enable evidence making from practice

A number of GRs described the importance of being strategic in their approach to grant applications. One GR saw grants as an opportunity to fill a knowledge gap:

I often use a different technique to leverage money, and that is to look at a particular issue and say, “Oh, I wish I better understood that particular problem.” I wonder if I can get $10,000 to do a little research post and then we’ve got the answer to that problem.

The grants programme allowed another GR to pursue an evidence-based programme where they felt necessary services were lacking:

We would go online and see what was being funded, and not necessarily agree with what was being funded. Then I think part of it was also just being fed up like, “What do we use to apply for this? We’re the best situated to deliver this.”

Though sometimes, even with long-running programmes demonstrated to work, GRs described how continued avenues for funding were not always forthcoming. In continually having to look for grants to fund successful programmes, one GR explained how they felt compelled to reinvent successful programmes so they could continue to attract money from grant competitions.

#### The risk of grants as a ‘drain’ on resources

For GRs working within small organizations, grant competitions could be ‘a double-edged sword’. As numerous GRs made clear, getting the grant money was only one step in effecting change:

Sometimes getting told you’re successful is scary… thinking, ‘Do we have the resources to make it successful for our community?’ I mean, at the end of the day, spending the money and having the grant is great, but it needs to make a difference in your community.

Additional resources—particularly staff time—were needed:

Having extra funding doesn’t mean that you’re going to get extra programs or better programs… because people have got to have the capacity to be able to run them.

As one GR described, ‘we have so few resources to help do anything other than our direct work’. So programmes funded through grants competitions could really stretch resources.

For organizations that had no recurrent funding, the continuous search for grant money was not only taxing, it was critical for service provision:

Any programs… or events that we want to run, or host, or facilitate, we actually have to find the funding for them… without the funding, we wouldn’t be having [the program]

To make funding applications competitive, one GR described keeping programme budgets very lean, minimizing requests for overhead costs. But this meant grants could be ‘a drain’ on small organizations. By contrast, larger organizations, including local government, usually had secure staffing arrangements and a larger workforce, so grant applications could just include ‘the bare bones of what you need to deliver’ programmes.

Many GRs emphasized the importance of a targeted approach to grant competitions, because there was a danger in taking on too much. One GR recalled how their organization had once applied for more grants than they could reasonably manage:

The team had put in for all these grants thinking they might get three and then they got eight… There was definitely some sense of that was way too much to do. We winged it, we should never do that again.

As well as illustrating the difficulties of managing too many grant-funded programmes, this shows how the lack of predictability around funding through grant competitions means that even with a targeted approach, grant-funded activities are difficult to plan around.

#### The challenge of finding funding for staff

It was often the case that funding did not provide the resources GRs felt were most needed. For those in smaller organizations, staffing issues were brought up over and over during interviews. Many lamented that there were not more large grants available to fund staff:

Sometimes [grant applications] are not worth writing because they don’t give you what you need. I mean, I’d love to have a whole other person. But if the grant’s only 70,000 or 80,000 I’m not applying for it because it’s not enough money. And I don’t want a part person, I want a whole person.

Another GR spoke about how great it was when they got funding for staff instead of relying purely on volunteers—but also made clear this was very rare. Their organization had calculated the value of their volunteers as far exceeding AU$1 million. Yet they felt too much was expected of volunteers:

There’s too much emphasis with funding bodies on everything has to be volunteered. You need to be able to recompense people something sometime… there’s a lot of money contributed in kind. And they do great work, it’s not like they’re sitting around taking up time, they’re actually contributing.

### Insights from Review Panel meetings

The Review Panel meetings offer an additional perspective on the grant competition as a mechanism to leverage resources in organizations within the wider prevention system. Proposals are assessed based on the potential of individual applications as well as what each application represents in terms of the overall pool of applicants. While the assessment process is shaped by predetermined eligibility criteria, panel discussions reveal how panel members try to ascertain which applicants will be the best ‘fit’ for the grant competition purpose. Applications that requested funding for ongoing operational costs or ongoing salaries were not eligible. Those requesting funds for staffing had to explain how this would leave lasting skills and knowledge within the community.

Panel discussions were supplemented by background work before and after the panel meetings. Findings from the research team’s analyses of the 2019 review panel meetings and GR interviews were provided to the 2020 research panel members prompting discussions around the benefits of funding for network/relationship building, how to balance applications from smaller and larger organizations and contemplating the meaning of ‘sustainability’ in the context of short-term grants. Background work was also necessary for panel members to keep abreast of other funding sources that align with or augment the activities proposed by applicants. One of the tasks facing the panel was, therefore, to discern whether the application was requesting funds for *new* activities or if they already received funding for proposed services (making them ineligible).

#### Sorting ‘core’ business from new initiatives

Panel members were wary of funding programmes that seemed to ‘prop up’ organizations or where ongoing funding would be needed to support a particular programme. They deliberated over whether applicants were requesting funds for core activities, and often questioned whether or not the applicant organization ought to be ‘doing this already’. A ‘positive’ proposal could be knocked out by suggestions that proposed activities should be part of an applicant organization’s ‘core business’.

But determining what ought to be ‘core’ for an applicant organization was not always straightforward, particularly with smaller organizations. When the panel discussed whether a local council should be funded for a community event, for example, the panel was divided. One panel member felt councils should be running events as a matter of course, rather than through grants. However, another panel member pointed out that small councils did not necessarily have funding to deliver on health and well-being initiatives. In this sense, the concept of ‘core’ activities was considered against whether smaller organizations could carry the infrastructure/overhead costs of a new programme, and also whether ‘health’ was an existing part of an organization’s mission.

Requests for staff wages were often a red flag for the panel, raising questions about the sustainability of the programme beyond the 2-year grant period. One application received initial favourable assessments based on good connections with relevant experts and effective engagement with high-risk groups. But when a panel member pointed out the budget relied heavily on staff wages, it was decided the proposed programme needed ongoing funding rather than a one-off grant. The issue of where such ongoing funds could potentially come from was not broached in this discussion.

#### Grants as ‘icing on the cake’

As panel members considered each application, they often grappled with the meaning and purpose of grants as a whole. A striking example was a discussion regarding an application to build kitchen equipment. The panel members wondered together whether this was appropriate for this grant competition, as it was not intended for infrastructure. It was raised that a kitchen could serve as ‘a vehicle’ for all kinds of important community connections. However, some felt the organization already had good engagement and community activities were already happening. The panel noted the application depended on funding from two other sources, both of which were listed as ‘pending’ and appeared not to be confirmed. For the panel, it was therefore ‘a risk’ to grant funding:

I think we’re going to have to say, ‘No,’ because what if those [other funding applications] ones don’t come off? Then they can’t put icing on the cake if the cake’s not there.

Here we see how the ‘icing on the cake’ metaphor, also used by GRs, conveys how panel members are looking for applicant organizations to have minimum resource structures in place before they can reasonably allocate extra funding through the grant competition See Table 1 for an overview of the impact of grant competitions on community-level resources.

**Table 1:** Summary of grant competition impact on community-level resources

What grants enable	The qualifiers
• Opportunity to experiment with new ideas (in stable, mature organizations) • Opportunity to clarify ideas and build momentum • Opportunity to orient an organization in a new direction (i.e. towards explicitly promoting health) • Stepping stones in building specific capacity • Opportunity to build on learnings from practice and collect evidence to verify the effectiveness of a new practice/programme and build a case for programme scale-up	• Need pre-existing inter- organizational relationships/partnerships to be successful • Too much is still expected from volunteers, even in the presence of funding • Small organizations do not have the capacity to put forward competitive ‘lean’ budget bids in compared to larger organizations • Small organizations don’t have capacity/staff to run ‘extra’ programmes even with funding • Writing grants and securing funding is taxing/often not worth it • Organizations try for several grants at once, and stress comes from being successful with all of them • Some grants may only be successful in the presence of other separate parallel grants/activities. Uncertainty about these discourages award of grants one at a time • What is really needed (core, full time staff) and what an organization can apply for do not match

## DISCUSSION

We note that our interviews were only held with successful grant recipients. However, many participants discussed their experiences being unsuccessful in other grant competitions. It is not unusual to interview grant receivers, but by using interview material alongside the deliberations of grant funding panels, this study provides a unique, real-time account of the merits of individual grants, as well as the larger context and dynamics in which decisions were being made. Having the ‘giver’ and ‘receiver’ data sets side by side enabled us to see how GRs and panel members often mirrored each other. For example, where GRs felt grants could be a drain on the resources of small organizations due to trimming the budget to keep down overhead costs, panel members were similarly wary of seemingly undercooked budgets that could indicate that an applicant organization was inexperienced or being overly ambitious.

We saw how funding could benefit organizations and we saw what else was needed to bring about broader change in communities. Many GRs acknowledged that getting a grant was valuable but making a difference in communities required much more than money. Significant ‘pre-application’ resources were required to plan a new programme of work. GRs from small organizations felt disadvantaged where they needed to factor in staff or overheads in their budgets, whereas larger organizations had the capacity to leave these ‘core’ costs out. Others have observed that competitive processes impede the ability of smaller organizations to respond to emerging social issues (Productivity Commission, 2010).

GRs described how grant funding enabled organizational development. As one GR explained, ‘it’s not all about the money’ but rather that money could ignite a symbolic shift for organizations through directing priorities towards a health and well-being agenda. Grants could also help build the organizational momentum and facilitate future grant success. This happened through building critical partnerships, or through creating relationships with funders, building ‘inside’ knowledge or building a reputation as a trusted, reliable organization. For some organizations, funding allowed for the development of novel ideas or new knowledge based on learnings from practice. Generating practice-based evidence was an important stepping stone for organizations wanting to scale new services in the community. However, these gains can potentially create a ‘privileged class of grant seeker’ as the application process favours those with social capital such as social networks and established legitimacy in the field (Holm, 2019, p. 220).

Both GRs and funders spoke of ‘icing’ and ‘cake’. The icing-on-the-cake metaphor is often understood to mean taking something that is good (cake) and making it even better (icing). We saw how the icing-on-the-cake metaphor provided a way for GRs and panel members to express an ideal-typical scenario for how the grants competition *ought* to work: by granting funds (icing) to organizations to enhance core work (cake) that they already do. This icing-cake model implies a clear delineation between core activities/resources of an organization and extras that would ideally be funded by grants programmes. For well-established organizations who have secured recurrent core funding to deliver prevention services, this model can work well to stimulate new programmes of work and experiment with new ideas. And yet the findings also reveal a gap between this ideal icing-cake model of grants competitions and what many organizations require to undertake long-term processes of change in communities. This gap is illustrated as GRs describe the challenges of getting funding for what they really need and the pressures of trying to carry out funded programmes of work with inadequate paid staffing roles. The gap is further evidenced by struggles of panel members as they assess applications that offer promise, but are not well served by short-term funding arrangements and the constraints that are typical of a categorical grant competition.

What the icing-cake metaphor really signals is a lack of balance in the overall mix of funding mechanisms for health promotion. There is a lack of fit in terms of what many organizations require and the larger system of funding support for local health promotion practice. The challenges described by organizations could not simply be solved by coming up with a better idea or improved application. Similarly, the review panel was faced with navigating questions about sustainability of grant proposals that required support for ‘core’ activities that were ineligible under the terms of the competition.

Many of the key elements of community capacity cannot be provided directly through external funding mechanisms. Instead, they must be built and maintained over time by community workers and volunteers. External funds are still helpful in developing the necessary soft infrastructure (e.g. hope, trust, self-efficacy, relationships, governance) but the funds must be provided in ways that accommodate the flexibility and varied timelines that capacity building requires (Kavanagh *et al*., 2020). Competitive grant funding can only ever be part of the equation (Kavanagh *et al*., 2022).

More work is needed to determine the ideal mix of ways to fund prevention. Recent initiatives have promoted community decision-making through collaborative community budgets (Kaszynska *et al*., 2012) and collective control of money to foster empowerment of communities as a route to addressing health inequalities (Townsend *et al*., 2020). In addition, we suggest considering a move towards a more explicit community-centred grant funding model which allows state-wide topics to be addressed while catering to a diversity of community abilities. As well as aiming to encourage activities and services that move communities further ahead in terms of well-being, community-centred grants would aim to progress local capacity according to the stage of community development need. For example, applicants could be streamed according to three pools of funding: (i) microgrants for capacity building/safe to fail experimentation, including very limited reporting requirements; (ii) grants to develop evidence or programme evaluation to prepare for scale-up; and (iii) commissioning fund for state-wide or long-term roll-out, ongoing support for ‘core’ activities.

This would allow for more realistic expectations of ‘sustainability’ criteria according to the funding pool. Programme sustainability (e.g. in terms of staffing) in the absence of continued funding (as in pools 1 and 2) should not be expected. Instead, a narrative about how the grant has made residual capacity better could be more appropriate. Applications could also be assessed with flexible timing, rather than having all applicants submit at the same time. This would have the advantage of suiting the community pace of change.

Some of these processes are already underway in our project, as the funding body has used findings from GR interviews to inform quality improvement. Recognizing the grant process favours those with experience and pre-existing resources (including grant-writing skills), the funding body has made grant application processes more accessible to a wider range of organizations. Measures such as plain language documents and user-friendly systems have been introduced. Efforts have been made to even the playing field through introducing forums, skill-building sessions, sharing experiences of successful applicants and practical support to help applicants move from an idea to application. Similar to Marsh and colleagues (2008), the funding body has developed a ‘partnership’ model whereby the funding process is viewed as a learning opportunity that can provide support with programme implementation and evaluation. For example, content advisors and grant recipients come together for forums to foster intelligence gathering and relationship building. Our research team is also working to investigate and restore better population-based ways of funding prevention so that the responsibility for capacity building at a community level does not fall to one-off granting schemes. Rather than tempt applicants to disguise or ‘dress up’ local capacity, grant ‘competitions’ should encourage disclosure of real-world circumstances and build capacity from the ground up.

## Supplementary Material

daac115_suppl_Supplementary_Appendix
