# Supplementary material for: Icing or cake? Grant competitions as a model for funding chronic disease prevention in Tasmania, Australia
Source: Health Promot Int. 2022 Sep 27;37(5):daac115. doi: 10.1093/heapro/daac115 (PMC9514227; doi:10.1093/heapro/daac115)
Supplement: daac115_suppl_Supplementary_Appendix [file daac115_suppl_supplementary_appendix.docx]

**Appendix 1 Interview schedule - grant recipients**

| 1 | Experience during COVID-19 | - How have things changed for your funded program/organisation during COVID? |
| --- | --- | --- |
| 2 | Grant Story | - Can you tell me a bit about your role within the organisation? - How long have you been working in the organisation? What experience did you have working with community organisations before that? - Can you tell me about how your organisation and the grant program is dealing with the current change in circumstances? (eg. Is support from [funding body] useful? What changes are happening: Changes to activities, timeframes reporting and funding) |
| 3 | Grant application history | - *First time/ repeat application to this funder? Did you apply for, or receive funding as part of the community innovations grants?* - How did you find out about it? - Same people involved this time/different? - Did the funding round prompt you to develop or shape an idea or was it well formed awaiting sources of funding? - Has same idea been put to other funders? Why? Why not? Outcome? - *So, a new application altogether? Or redeveloped/recycled one?* - Did you change the size of what you wanted to do to fit the funding amount? How? - If they have received funding in the past then: how well do you feel you were able to work within the amount you requested for funding? Did you adjust your budget based on what you learned from past projects and how much was required to carry out the program? |
| 4 | Value add and fit with existing activity | - What does this grant add relative to what you are already doing in this area? (i.e., what doing with this $ that you could not otherwise do) - Why is that important? - *Does it free up resources that will now be allocated elsewhere? Yes/No. Or does it add existing resources that will all be devoted to the same thing now? Yes/No. Explain.* - Does funding create short term positions that in themselves lead to other ‘needs’ ? eg. need to renewal etc, recruitment problems - How has this grant affected your ability to adapt to the current circumstances in other work that you are doing? |
| 5 | Preparation and time taken | - *When started working on application (relative to deadline) in days/weeks?* - *How many people (name), what role each took, hours/days per person (to be recorded in a table)?*   *eg,*  *Name Role Time in hours/days*  *Sally Main writer 3 full days, a weekend (2 full days, and one Saturday night)*  *1 day editing and filling out form*  *Chia Literature 1 day*  *Ahmed Feedback/edit 4 hours* |
| 6 | Fit with the way organisation is currently funded | - *What proportion of (this organisation’s) activity is grant funded?* - *How many grants per year would you be applying for? How many staff would be in taking the lead role (writer?)* - *What is your success rate, roughly?* |
| 7 | Capacity uplift | - We often hear that you have to have money/resources in order to attract money/resources – is that true here? How? - *Are there some types of resources or capacity you feel you need that funds/grants can’t buy? Yes/No. Explain.* - For Large Grant recipients: Have you received a small grant in the past? Are there ways that the smaller grants were able to help you build evidence to then apply for large grants? - Can getting grant funds be a burden as well as enabler? How? Give examples. - Can being unsuccessful still have benefits? Explain. |
| 8 | Organisational support from [funding body] | - Have you participated in the Network forums organised by [funding body]? Were you able to build on resources that you learned about within other organisations and/or make connections? - Have you seen the grant stories on the [funding body] web portal? Have you been able to make use of these for building your organisational capacity? Has it been useful for obtaining future grants? |
| 9 | Private fee-for-service consultants | - Check if they use them. If yes then: - What gap is this filling? Is there a way that you think you could build that capacity within your organisation? What kind of funding support would that require? - How much do such consultants charge? Do applicants think this is reasonable or good value for money? How? Why? By what criterion? What amount of $ would make the use prohibitive? |
| 10 | Hitting targets: singly or in clusters and tangents | - Funding is often designed to address one key priority, but in the field programs work less directly. Practitioners find that to achieve “X” with a target group they might have to address “Y” or “Z” first. Do you have any examples of that? - How does it affect the way you work? Because in reality you are being pressed to deliver on “X” only? |
